# Angiotensin Receptor Blockers for COVID-19: Pathophysiological and Pharmacological Considerations About Ongoing and Future Prospective Clinical Trials

**DOI:** 10.3389/fphar.2021.603736

**Published:** 2021-03-29

**Authors:** Rodolfo Pedro Rothlin, Mariano Duarte, Facundo Germán Pelorosso, Liliana Nicolosi, M. Victoria Salgado, Héctor Miguel Vetulli, Eduardo Spitzer

**Affiliations:** ^1^ Sociedad Argentina de Farmacología Clínica, Asociación Médica Argentina, Buenos Aires, Argentina; ^2^ Hospital de Clínicas ‘José de San Martín’, Facultad de Medicina, Universidad de Buenos Aires, Buenos Aires, Argentina; ^3^ Hospital de Alta Complejidad El Calafate SAMIC, El Calafate, Provincia de Santa Cruz, Argentina; ^4^ Hospital Español de Buenos Aires, Buenos Aires, Argentina; ^5^ Centro de Estudios de Estado y Sociedad, Buenos Aires, Argentina; ^6^ Servicio de Electrofisiología Cardíaca, Arritmias y Marcapasos. Sanatorio Otamendi y Miroli, Buenos Aires, Argentina; ^7^ Laboratorio Elea Phoenix S.A, Buenos Aires, Argentina

**Keywords:** angiotensin receptor blocker, clinical trial, telmisartan, valsartan, losartan, COVID-19

## Abstract

COVID-19 pandemic demands a swift response to find therapeutic tools that effectively reduce morbidity and mortality. Despite initial fears, evidence from retrospective observational studies supports the inhibition of the renin-angiotensin system as an emerging pathway to delay or moderate angiotensin II-driven lung inflammation. This has triggered several prospective clinical trials. In this commentary we provide an overview and analysis of current ongoing clinical trials aimed at evaluating the therapeutic efficacy of angiotensin receptor blocker (ARB) use in COVID-19. The relevance of the results of these trials will have to be interpreted depending on the stage and severity of the disease and in light of the start time of their prescription related to the time of diagnosis of COVID-19 as well as the administered doses.

## Introduction

In the late 1930s, two groups competed in a memorable race: One led by Braun Menendez and backed by Bernardo Houssay (Nobel Prize in Physiology or Medicine 1947) at the Physiology Department of the School of Medicine of the University of Buenos Aires (Argentina) and the other led by Irvine Page at Lilly Research Laboratories in Indianapolis. The two research groups, working independently, reached similar conclusions. The argentine group postulated that an enzyme-substrate type of reaction involving a substance they named hypertensin explained ischemic kidney-related hypertension (Braun-Menendez et al., 1940) while Page and his group proposed that there was a plasma activator of renin that resulted in the production of a crystalline pressor substance, which they called *angiotonin* (Page and Helmer, 1940). Braun Menendez and Page eventually agreed on the hybrid term angiotensin (Braun-Menendez and Page, 1958). More than sixty years later, angiotensin is brought again to the center stage by de SARS-CoV-2 pandemic (Gurwitz, 2020).

The renin-angiotensin system (RAS) is crucial in vascular, cardiac, and renal physiology and is one of the most important participants in both short-term and long-term regulation of blood pressure. Renin is an enzyme (aspartyl protease) that attacks an abundant circulating α2 globulin, angiotensinogen, to catalyze the formation of the decapeptide angiotensin I. This decapeptide is then cleaved by angiotensin converting enzyme (ACE) giving rise to the formation of octapeptide angiotensin II (Ang II). This happens mostly in the lung largely due to the activity of membrane-bound ACE present on the luminal aspect of endothelial cells throughout the vascular system. Ang II is transformed by ACE2 to Ang 1–7. Angiotensin II acting on AT1 receptors causes vasoconstriction, apoptosis, proinflammatory effects, and fibrosis and, acting through AT2 receptors, has anti-inflammatory and antifibrotic effects in different tissues (Wang et al., 2017). In lung, this effect may be of significance in patients with COVID-19. Angiotensin 1–7 acting on Mas receptors causes opposite effects: vasodilation and anti-inflammation. In the traditional view of the RAS, Ang II is delivered to its target organs via the blood stream but, in the last years, growing evidence of the presence of local (tissue) RAS has been reported, demonstrating multiple synthesis sites of Ang II including vascular endothelium, heart, kidney, brain, adipose tissue, liver, adrenal gland, and even gonads and placenta (Paul et al., 2006).

Coronavirus disease 2019 (COVID-19) is caused by the SARS-CoV-2 virus that enters the airway and binds the host cell (alveolar type 2) through the interaction of the structural protein S (spike) with the protein membrane ACE2 (angiotensin converting enzyme 2) (Wan et al., 2020). The virus–ACE2 complex is internalized by endocytosis effectively sequestering (apparent downregulation) ACE2 which in turn loses its function catalyzing the degradation of angiotensin II to angiotensin 1–7. Partial decrease or total loss of ACE2 function in alveolar cells results in a deviation of the homeostatic balance of the renin-angiotensin system in favor of the angiotensin II–AT1 receptor axis (Tikellis et al., 2011; Paz Ocaranza et al., 2020).

Taking into account previous studies on animals that have shown that chronic treatments with ACEI/ARBs could upregulate circulating ACE2 (while clinical studies had failed to demonstrate such an association), it was postulated at the beginning of the COVID-19 pandemic that treatment with ACEIs/ARBs could be harmful, could affect SARS-CoV-2 infectivity, and may alter COVID-19 disease progression by altering ACE2 expression (Esler and Esler, 2020; Fang et al., 2020). Subsequently, Sriram and Insel published a literature review of studies in experimental animals (n = 12) and human subjects (n = 12) and evaluated the evidence regarding the impact of administration of ACEIs and ARBs on ACE2 expression. These authors conclude that the current evidence, especially from human studies, does not support the idea that treatment with ACEIs or ARBs produces pathophysiological relevant increases in ACE2 protein abundance (Sriram and Insel, 2020).

Despite significant expression of ACE2, in the year 2003, there was no dimension of the importance of its protective functions in the lung until the appearance of serious respiratory forms of coronavirus infection called SARS (Severe Acute Respiratory Syndrome) (Ksiazek et al., 2003). At that time, it was found that the protein S (spike) on the surface of the virus allowed the microorganism to enter the host’s lung using ACE2 as a receptor in the surface of type II pneumocytes (Hamming et al., 2004; Kuba et al., 2005) and downregulation of ACE2 expression was observed using both experimental SARS-CoV infections of wildtype mice *in vivo* in the lungs and recombinant SARS-CoV surface-spike protein binding to ACE2 in cell lines (Kuba et al., 2005). Treatment with spike protein worsened the lung function in wildtype mice but did not affect the severity of lung failure in Ace2 knockout mice, indicating that the effect of spike protein on acute lung injury is ACE2 specific (Kuba et al., 2005). Furthermore, Kuba et al. showed that an AT1 receptor blocker (losartan) attenuated acute severe lung injury and pulmonary edema in spike-Fc treated mice. The authors conclude that their data provide a molecular link between SARS pathogenesis and the role of renin-angiotensin system in lung failure. Recently, Patra et al. (Patra et al., 2020) showed that SARS-CoV-2 infection or ectopic spike protein expression in human epithelial cells inhibited ACE2 expression, leading to increased angiotensin II and AT1 receptor expression. They also demonstrated that SARS-CoV-2 spike protein was associated with the upregulation of AT1 signaling which led to the induction of transcriptional regulatory molecules, such as NF-κB, c-Fos, and MAPK activation. Spike protein induced the generation of IL-6 in cultured cells as well as in COVID-19 positive patient sera, and AT1 receptor antagonist (candesartan cilexetil) resulted in downregulation of MAPK activation as well as IL-6 release.

Clinically, patients with severe coronavirus disease 2019 (COVID-19) have labored breathing and progressive hypoxemia and often receive mechanical ventilatory support. Radiographically, peripheral lung ground-glass opacities on computed tomographic (CT) imaging of the chest fulfill the Berlin criteria for acute respiratory distress syndrome (ARDS) (Raptis et al., 2020). The histologic correlate of ARDS is widely considered to be “diffuse alveolar damage,” a continuum of changes constituted by the rapid development of capillary congestion, atelectasis, intra-alveolar hemorrhage, and alveolar edema, followed days later by hyaline-membrane formation, epithelial-cell hyperplasia, and interstitial edema (Thompson et al., 2017). The lungs from the patients with COVID-19 show a morphologic pattern of diffuse alveolar damage and infiltrating perivascular lymphocytes. In addition, there are distinctive angiocentric features of COVID-19, characterized by severe endothelial injury associated with intracellular SARS-CoV-2 virus and disrupted endothelial cell membranes; widespread vascular thrombosis with microangiopathy and occlusion of alveolar capillaries; and significant new vessel growth through a mechanism of intussusceptive angiogenesis (Ackermann et al., 2020).

It has been hypothesized that this inflammatory process depends on the stimulation of AT1 receptors by Ang II without the counter-regulatory catalytic activity of ACE2 (Gurwitz, 2020; Saavedra, 2020). Accordingly, repurposing of ARBs has been proposed for the tentative treatment of COVID-19 patients (Gurwitz, 2020).

## Angiotensin Receptor Blockers in COVID-19

### Retrospective Studies

There is relevant information from retrospective studies in patients with COVID-19, evaluating the susceptibility, severity, and mortality of this disease in patients treated with ACE inhibitors (ACEi)/ARB. Recently, a meta-analysis has been published analyzing whether the use of ACEi/ARB as antihypertensive drugs was associated with severity and mortality in patients with COVID-19 which included nine studies involving 3,936 patients. Antihypertensive treatment with ACEi/ARB, compared to non-ACEI/ARB treatment, was not associated with disease severity but was linked to lower mortality (Guo et al., 1979). Also, another recent meta-analysis showed that among 24,676 COVID-19 patients, overall assessment by estimating random effects shows that the use of ACEIs/ARBs is not associated with higher risk of in-hospital death and/or severe illness among hypertensive patients with COVID-19 infection. On the contrary, effect estimate shows an overall protective effect of RAAS inhibitors/blockers (Pirola and Sookoian, 2020).

A recent retrospective analysis showed that ACEi, but not ARBs, were associated with a significant lower risk of hospitalization in a subgroup of patients (Khera et al., 2020). In addition, meta-analysis of retrospective studies shows that blocking the RAAS may decrease all-cause mortality in COVID-19 patients (Chu et al., 2020).

### Prospective Studies

Main prospective studies on ARBs and COVID-19 were surveyed with https://www.clinicaltrials.gov/ using the search string “angiotensin receptor blocker” and “covid” in the disease term field. Studies fulfilling these criteria involve losartan, valsartan, and telmisartan. Losartan is a competitive AT1 receptor blocker and presents an affinity estimate (pKi or pIC50) determined in competition radioligands binding studies at mammalian AT1R of 7.71. Approximately, 14% of an oral dose of losartan is converted to the 5-carboxylic acid metabolite, EXP3174, which is more potent than losartan as an AT1 receptor antagonist (affinity estimates at mammalian AT1R: 8.17). Affinity estimates of valsartan and telmisartan are at mammalian AT1R: 8.46 and 8.33, respectively (Michel et al., 2013). Furthermore, insurmountable antagonism (the maximum effect of an agonist is reduced by pretreatment with or presence of the antagonist) is a property that almost all ARBs have to some degree and appears to be a direct function of dissociation rate from receptor in kinetic radioligand binding studies. Using cloned human AT1R, the dissociation half-lives from the receptor exhibited an order of telmisartan > valsartan ∼ losartan (213, 70, and 67 min, respectively) (Kakuta et al., 2005). Pharmacokinetic properties: losartan has an oral bioavailability of approximately 33%, a volume of distribution (Vd) of 34 L, a high binding to plasma proteins of approximately 99%, and a plasma elimination half-life of 2 h. Valsartan has an oral bioavailability of approximately 25%, a Vd of 17 L, a binding to plasma proteins of 95%, and a plasma half-life of 6 h. Telmisartan has an oral bioavailability of 42–58%, a Vd of 500 L, a binding to plasma proteins greater than 99.5%, and a plasma half-life of 24 h (Michel et al., 2013).

### Losartan

In relation to the prospective clinical trials currently using ARBs and registered at www.ClinicalTrials.gov (accessed on 08-25-2020), it is worth mentioning NCT 04311177 (Table 1) which excludes patients with an onset of symptoms prior to 7 days before randomization and evaluates losartan in COVID-19 patients without respiratory distress for 10 days albeit at low doses (25 mg/day).

**TABLE 1:** Main characteristics of losartan and valsartan prospective clinical trials on COVID-19.

Trial ID	NCT04311177	NCT04312009	NCT04328012	NCT04340557	NCT04335786
Agent	losartan	losartan	losartan	losartan	valsartan
Dosing (daily)	25 mg	50 mg	25 mg	25 mg	80–160 mg
Duration of treatment	10 days	7 days or until hospital discharge	5–14 days	10 days	14 days
Treatment initiation	Within 72 h of meeting inclusion criteria and within 7 days of symptom onset	Within 48 h of presentation or hospital admission or within 48 h of a positive test result	Within 72 h of SARS-CoV-2 infection diagnosis	Confirmed SARS-CoV-2 positive test result	Within 24 h of confirmed in-hospital SARS-CoV-2 infection diagnosis
Severity at admission	Outpatient setting. Upper respiratory symptoms (cough, rhinorrhea) or fever ( > 101.5) or loss of taste/smell	Admission to the hospital with a respiratory SOFA≥1 and increased oxygen requirement compared to baseline among those on home O2	Hospitalized patient	Mild to moderate respiratory symptoms of COVID-19. Excluded if in the intensive care unit at screening	Admitted to the hospital but not in ICU
Primary endpoint	Hospital admission within 15 days	Difference in estimated (PEEP adjusted) P/F ratio at 7 days	National Institute of Allergy and Infectious Diseases COVID-19 Ordinal Severity Scale (NCOSS) within 60 days	Mechanical ventilation	First occurrence of intensive care unit admission, mechanical ventilation or death within 14 days
Estimated enrollment	580	200	4000	200	651
Allocation	Randomized	Randomized	Randomized	Randomized	Randomized
Intervention model	Parallel Assignment. Placebo controlled clinical trial	Parallel Assignment. Placebo controlled clinical trial	Parallel Assignment. Placebo controlled clinical trial	Parallel Assignment Intervention	Parallel Assignment. Placebo controlled clinical trial
Masking	Quadruple (Participant, Care Provider, Investigator, Outcomes Assessor)	Quadruple (Participant, Care Provider, Investigator, Outcomes Assessor)	Quadruple (Participant, Care Provider, Investigator, Outcomes Assessor)	None	Quadruple (Participant, Care Provider, Investigator, Outcomes Assessor)
Ages eligible for study	18 years and older	18 years and older	18 years and older	18 years and older	18 years and older
Sexes eligible for study	All	All	All	All	All

The remaining trials with losartan (NCT04312009, NCT04328012, NCT04340557, Table 1) are characterized by being applied in patients with COVID-19 hospitalized for lung injury within 48 to 72 h of a positive SARS-CoV-2 test and with different degrees of respiratory failure and at doses also lower than the maximum antihypertensive of 100 mg/day.

### Valsartan

NCT04335786 (Table 1) evaluates valsartan in patients enrolled within 24 h of a positive SARS-CoV-2 test in initial doses of 80 mg b.i.d. and escalating to maximal antihypertensive dose of 160 mg b.i.d. for 14 days in hospitalized patients with lung involvement but not in intensive care unit (ICU) (Gommans et al., 2020).

### Telmisartan

Telmisartan is being evaluated in NCT04355936 (Rothlin et al., 2020; Table 2). This ARB was chosen for its pharmacological properties and is evaluated for 14 days in hospitalized patients with COVID-19 but not in ICU and within four or less days of symptoms before enrollment. Considering that the adverse effects of this agent have been described as placebo like (Schumacher and Mancia, 2008), the doses used are 80 mg b.i.d. (corresponding to twice the maximum antihypertensive recommended dose).

**TABLE 2:** Main characteristics of telmisartan prospective clinical trials on COVID-19.

Trial ID	NCT04355936	NCT04356495	NCT04359953	NCT04360551	NCT04510662
Agent	Telmisartan	Telmisartan	Telmisartan	Telmisartan	Telmisartan
Dosing (daily)	160 mg	20 mg	80 mg	40 mg	40 mg
Duration of treatment	14 days	10 days	14 days	21 days	14 days
Treatment initiation	Positive SARS-CoV-2 test and illness symptoms beginning 4 days or less prior to randomization	Onset of symptoms <5 days prior to nasopharyngeal swab sampling	Positive SARS-CoV-2 test and less than 5 days from symptom onset to randomization	Within 72 h of a laboratory-confirmed severe acute respiratory syndrome corona virus 2 (SARS-CoV-2) infection as determined by FDA-approved commercial or public health assay	Within 24 h of diagnosis
Severity at admission	Hospitalization for COVID-19 but not in ICU	Outpatient setting	Clinical manifestation of COVID 19 requiring hospitalization: pneumopathy and/or upper airway infection and/or respiratory distress, confusion and/or encephalopathy and/or signs of encephalitis, walking disorders with ataxia and/or falls, digestive problem (diarrhea and/or vomiting)	Outpatient setting	Admitted to the hospital but not in ICU. Hypoxic respiratory failure: SpO2 ≤94% on room or tachypnea (respiratory rate ≥22 breaths/min)
Primary endpoint	C-reactive protein levels at days 5 and 8 after enrollment	Proportion of participants with an occurrence of hospitalization to day 14. Proportion of participants with an occurrence of death to day 14	Two-week survival rate	Maximum clinical severity of disease within 21 days	Death within 30 days. Mechanical ventilation within 14 days
Estimated enrollment	400	634	1600	40	60
Allocation	Randomized	Randomized	Randomized	Randomized	Randomized
Intervention model	Parallel Assignment	Parallel Assignment	Parallel Assignment	Parallel Assignment. Placebo controlled clinical trial	Parallel Assignment
Masking	None	None	None	Triple (Participant, Care Provider, Investigator)	None
Ages eligible for study	18 years and older	50 years and older	60 years and older	18 years and older	18 years and older
Sexes eligible for study	All	All	All	All	All

Recently, preliminary results from NCT04355936 have been communicated showing that telmisartan decreased plasma C-reactive protein levels rapidly and in a sustained manner in hospitalized COVID-19 patients. Moreover, telmisartan treatment produced a significative improvement in the clinical evolution of patients hospitalized with COVID-19 as evidenced by a shorter time to discharge (median time to discharge: 15 days in control group vs 9 days in telmisartan group). In the same line, patients still hospitalized at day 15 were less likely to require oxygen supplementation in the treated group than in the control group (Duarte et al., 2020).

Three additional clinical trials are ongoing employing telmisartan at low doses (NCT04356495, 20 mg/day for 10 days in patients with less than 5 days of symptoms before a positive SARS-CoV-2 test; NCT04360551, 40 mg/day for 21 days in patients with a positive SARS-CoV-2 test no more than 72 h before randomization; NCT04510662, 40 mg/day for 14 days in patients within 24 h of a positive SARS-CoV-2 test; Table 2) and another is employing telmisartan at maximal antihypertensive recommended dose for 14 days (NCT04359953, 40 mg b.i.d. in patients within 5 days of a positive SARS-CoV-2 test; Table 2).

## Discussion

An initial consequence of the proinflammatory stimulation of Ang II is the increase in the vascular permeability that leads to interstitial edema. It is possible that the clinical efficacy of the application of an ARB may be conditioned by the lapse between the start of the inflammatory process induced by the SARS-CoV-2 and the moment of its administration. In an analogous manner to edema induced by local histamine application, which can be antagonized only by pretreatment with a H1 receptor blocker but not after histamine action has ensued (Skidgel and Erdos, 2005), timing of ARB administration might be a key in their possible efficacy in COVID-19. However, the process of pulmonary inflammation is complex and with the participation of several mechanisms of innate and acquired immunity but possibly remains motorized by the permanent stimulation of Ang II. In this sense, the design of a clinical trial using ARB should consider the pathophysiological aspects described. Probably, the early and sustained administration of an ARB will be more effective against COVID-19 than its late application in severe cases of lung injury.

The history of L-DOPA in patients with Parkinson Disease (PD) is illustrative of the importance of the dose of a drug in causing or not a therapeutic effect. After experimental studies O. Hornykiewicz concluded that the deficiency of dopamine in the basal ganglia constituted the cause of the extrapyramidal symptoms (akinesia, rigidity, and tremor) (Hornykiewicz, 2017). In 1961, W. Birkmayer administered l-DOPA i.v. in doses of up to 150 mg in PD patients and the effect in the first patient was so stunning (complete abolition or substantial relief of akinesia) that Hornykiewicz called it the “l-DOPA miracle” (Hornykiewicz, 2017). For several years, l-DOPA administration was not considered an adequate tool to treat patients with PD. Just in 1967, G. Cotzias introduced l-DOPA into clinical routine practice, and the “Cotzias regimen,” which is basically still used, converted the short-lasting i.v. l-DOPA (low doses) antiparkinsonian effect into a sustained improvement by oral l-DOPA (high doses; up to 16 g per day) (Cotzias et al., 1967).

A competitive antagonist agent of any type of receptor will demonstrate its potency and efficacy as a function, on one hand, of the biophase concentration of the agonist and, on the other hand, of the its effective doses. This is classically demonstrated by *in vitro* experiments, with isolated organ technique, executing agonist dose-response curves in absence and in presence of several different concentrations of antagonist, thus obtaining values of potency and efficacy of the blocker.

It has been shown that plasma angiotensin II (Liu et al., 2020) and aldosterone (Villard et al., 2020) levels are markedly elevated and are correlated with severity in COVID-19 patients. These findings add support to the rationale of a high dose approach for ARBs in COVID-19. This high dose scenario is possible by the safety profile of this therapeutic class; ARBs are generally well tolerated, with no known class-specific adverse events (Schumacher and Mancia, 2008). In this sense, the comparison with the effects of endogenous histamine on chronic urticaria and the efficacy of H1 antihistamines in relation to the dose is once again relevant. Second-generation H1 antihistamines (loratadine, cetirizine, etc.) are the drugs of choice for initial therapy and the dose can be increased up to fourfold the recommended dose (Zuberbier et al., 2018).

In sum, in relation to the hypothesis that involves the entry mechanism of SARS-CoV-2 in the lung and the RAS imbalance in favor of the proinflammatory effects of Ang II stimulating AT1 receptors, we consider that the results of the clinical trials described using ARBs will have to be interpreted depending on severity and stage of disease and in light of the start time of their prescription related to the time of diagnosis of COVID-19 as well as the administered daily dose and duration of treatment. Of these, their defined and approved therapeutic ranges should be considered, mainly in their use as antihypertensive drugs.
